# CDC20 regulates sensitivity to chemotherapy and radiation in glioblastoma stem cells

**DOI:** 10.1371/journal.pone.0270251

**Published:** 2022-06-23

**Authors:** Diane D. Mao, Ryan T. Cleary, Amit Gujar, Tatenda Mahlokozera, Albert H. Kim

**Affiliations:** 1 Department of Neurological Surgery, Washington University School of Medicine, St. Louis, Missouri, United States of America; 2 Department of Neurological Surgery, Saint Louis University School of Medicine, St. Louis, Missouri, United States of America; 3 The Jackson Laboratory in Genomic Medicine, Farmington, Connecticut, United States of America; 4 The Brain Tumor Center, Siteman Cancer Center, Washington University School of Medicine, St. Louis, Missouri, United States of America; Emory University, UNITED STATES

## Abstract

Glioblastoma stem cells (GSCs) are an important subpopulation in glioblastoma, implicated in tumor growth, tumor recurrence, and radiation resistance. Understanding the cellular mechanisms for chemo- and radiation resistance could lead to the development of new therapeutic strategies. Here, we demonstrate that CDC20 promotes resistance to chemotherapy and radiation therapy. CDC20 knockdown does not increase TMZ- and radiation-induced DNA damage, or alter DNA damage repair, but rather promotes cell death through accumulation of the pro-apoptotic protein, Bim. Our results identify a CDC20 signaling pathway that regulates chemo- and radiosensitivity in GSCs, with the potential for CDC20-targeted therapeutic strategies in the treatment of glioblastoma.

## Introduction

Glioblastoma is the most common primary malignant brain tumor in adults. Surgical resection followed by concurrent chemotherapy and fractionated radiation therapy is the current standard of care [1]. Despite advances in treatment, prognosis remains poor due to resistance to chemoradiation [2]. The genetic and epigenetic intratumoral heterogeneity of glioblastomas has been well described and is in part responsible for treatment resistance [3–6]. One particular subpopulation of tumor cells known as glioblastoma stem cells (GSCs) has garnered particular attention [7]. GSCs have been implicated in overall tumor growth, radiation resistance, and tumor recurrence [7–12].

The anaphase-promoting complex (APC) E3 ubiquitin ligase and its co-activator CDC20 (CDC20-APC) are critical for mitotic transition [13]. In addition to its role in cell cycle regulation, CDC20-APC has non-mitotic functions in the developing mammalian brain, including regulation of dendritic morphogenesis and pre-synaptic differentiation [14–16]. Recent studies have elucidated the importance of CDC20-APC in the maintenance of the aberrant developmental state of GSCs [12, 17]. CDC20-APC is required for GSC self-renewal and invasiveness, and CDC20 overexpression enhances tumor growth in vivo [12]. But whether CDC20-APC contributes to treatment resistance in GSCs is not known.

Here we report that CDC20 promotes resistance to both temozolomide (TMZ) and ionizing radiation and that CDC20 inhibition can sensitize GSCs to these therapies. These results demonstrate the important role of CDC20 in glioblastoma resistance to chemotherapy and radiation therapy, highlighting a potential therapeutic target to improve the efficacy of standard of care treatments.

## Methods

### Institutional approvals

All human study research related to this study has been approved by the Institutional Review Board (IRB #201211019 and #201409046, Washington University School of Medicine). All participants donating tissue signed informed consent prior to tissue banking. Our animal protocol (#21–0083) adheres to NIH and American Association for Laboratory Animal Science (AALAS) guidelines and has been approved by our Institutional Animal Care and Use Committee (IACUC).

### Antibodies and drugs

Antibodies used for this study include rabbit polyclonal anti-CDC20 (H-175) (Santa Cruz Biotechnology), rabbit monoclonal anti-Bim (C34C5) (Cell Signaling Technology), rabbit polyclonal anti-p21 antibody (ab227443) (Abcam), mouse monoclonal anti-α-tubulin antibody (Sigma), and rabbit monoclonal anti-phospho-histone H2A.X (Ser139) (Cell Signaling Technology). Temozolomide was purchased from Tocris Bioscience.

### Lentiviral transduction and shRNA

Lentiviral production and transduction methods have been previously described [12]. In brief, lentiviral transduction was performed by adding virus with 2 μg/ml polybrene for 4 hours to cells. Human gene target-directed shRNA plasmid (in pLKO.1) from the RNAi Consortium was used: *CDC20* RNAi targeting 5’-TGGTGGTAATGATAACTTGGT-3’ (Washington University RNAi Core). For both adherent and sphere culture, GSCs were first transduced in adherent format with CDC20 RNAi or control SH002 RNAi lentivirus 1 day after plating. 2 days after infection, cells were selected in 2 μg/ml puromycin for 5 days and then counted (Trypan blue) and passaged to either adherent or sphere format as indicated. The RNAi-resistant CDC20 rescue mutant in the lentiviral N103 vector has been previously described (12). For RNAi rescue experiments, CDC20-Res viruses were transduced into cells 2 days following transduction with RNAi viruses and then selected in puromycin for 5 days.

### Cell culture

The generation of human GSC cultures has been described previously [18]. In brief, tumor samples obtained directly from surgery were dissociated by mincing and incubation in Accutase (Sigma-Aldrich) for 20–60 min at 37C. Cell suspensions were passed through a 70-μm cell strainer (Falcon) and plated using NeuroCult NS-A Basal medium (Stem Cell technologies INC) with N2 and B27 supplement (Life Technology) for adherent cells and DMEM/Ham’s F12 (Gibco) with Glutamax and B27 without vitamin A supplement (Life Technology) for neurosphere culture. Murine EGF and Human FGF-2 (Peprotech INC), each at 20 ng/ml, were added to culture media for adherent and neurosphere cultures. Neurosphere GSCs were grown on ultra-low attachment dishes, and adherent GSCs were grown on polyornithine- and laminin- (Sigma-Aldrich) coated Primaria dishes/flasks (BD Biosciences). Media were replaced with half fresh GSC media every 2–3 days. Cells were routinely used between passages 5 and 20. Informed consent was obtained from patients for use of human tissue and cells, and all human tissue-related protocols used in this study were approved by the Institutional Review Board (Washington University). HEK293 cells were cultured in DMEM with 10% fetal bovine serum (FBS) and penicillin/streptomycin (Life Technologies). All cell lines were incubated at 37°C with 5% (vol/vol) CO_2._

### Measurement of cellular proliferation/viability

Adherent culture or neurosphere GSCs first were transduced with CDC20 RNAi or control SH002 RNAi in adherent format as detailed above, selected with puromycin 2 days later for 5 days, and then plated in either routine or nonadherent 96-well plates (for adherent and sphere cultures., respectively) and treated with 50 μM temozolomide (TMZ) or vehicle for 10 days. Cellular proliferation of adherent GSC lines was assessed using 3-(4,5-dimethylthiazol-2-yl)-5-(3-carboxymethoxyphenyl)-2-(4-sulfophenyl)-2H-tetrazolium (MTS, Promega) per manufacturer’s instructions. Cellular viability of neurosphere GSC lines was assessed by measuring the amount of ATP generated by viable cells using the CellTiter-Glo luminescent cell viability assay (Promega) per manufacturer’s instructions. MTS-reducing activity and luminescent ATP was normalized for each condition to control scrambled RNAi or vector lentiviruses as appropriate (100%). Experiments were performed in 3 or more wells per experiment in three to five independent experiments.

### Ionizing radiation treatment

Adherent GSCs were transduced with CDC20 RNAi or control SH002 RNAi and two days later were selected in puromycin for 5 days. Seven days post-transduction, cells seeded at defined cell densities according to radiation dose were plated and allowed to attach overnight. The next day, cells were irradiated with 0, 2, 4, or 6 Gy. Two weeks later, colonies were stained with 0.5% cresyl violet, and the number of colonies was counted manually in a blinded fashion. Counts represent surviving fraction relative to control. For CDC20 RNAi rescue experiments, normalized colony counts (Control = 100%) were performed in blinded fashion 2 weeks after ionizing radiation.

### Immunocytochemistry for γ-H2AX foci

Cells were fixed in 4% (wt/vol) paraformaldehyde and then washed in PBS. Cells were then permeabilized with 0.4% (vol/vol) Triton X-100 (Fisher Scientific) for 15 min, washed in PBS, then blocked in blocking solution (PBS containing 5% (vol/vol) normal goat serum (Vector laboratories), 2.5% (vol/vol) fetal bovine serum (Gibco), 3% (wt/vol) bovine serum albumin (Sigma)) for 30 min at room temperature. Immunostaining for γH2AX was then carried out using rabbit anti-phospho-histone H2A.X (Ser139) antibody for 2 hours at room temperature. Cells were then washed with PBS and incubated with appropriate secondary antibody conjugated to fluorescent dye Alexa Fluor 568 Goat Anti-Rabbit (Life Technologies) for 60 min at room temperature. After additional PBS washes, nuclei were stained with Hoechst 33342 (Invitrogen). Fluorescent images of the cells were taken using an automated inverted microscope, and the number of γH2AX foci was counted to determine mean foci per cell.

### Cell death measurements

GSCs were transduced with CDC20 RNAi or control Sh002 RNAi and then selected in puromycin 2 days later. Seven days post-transduction, cells were plated in a black 96-well plate (Corning) at a density of 5000 cells/well. The next day, cells were either irradiated with 0 or 4 Gy, or treated with vehicle or 200 μM TMZ for 24 hour. Cells were then labeled with YOYO-1 Iodide green fluorescent dye (1:10,000, Life technologies) for 6 days. Culture media containing 200 μM TMZ or vehicle and YOYO-1 was exchanged every 2 days. At day 6 nuclei were stained with Hoechst 33342 (Invitrogen) 1:5000 in PBS for 5 minutes. Both YOYO-1 green cells and blue nuclei were imaged by inverted fluorescent microscope (Leica) at 20X magnification. ImageJ software was used to count YOYO-1^+^ cells and all cells (Hoechst 33342).

### Statistics

All images are representative of results from at least three independent experiments. Statistical analyses were performed with Excel (Microsoft) or Prism (Version 9.1.2) software. The unpaired Student’s t test was used for comparisons in experiments with only two groups. In experiments with more than two comparison groups, ANOVA was performed followed by Fisher’s least significant difference or the Bonferroni test for pairwise comparisons among three and greater than three groups, respectively.

## Results

We previously generated low-passage, patient-derived glioblastoma cell lines using defined serum-free media [12, 18]. These primary glioblastoma cells, also referred to as glioblastoma stem cells (GSCs), express neural stem cell markers and exhibit self-renewal *in vitro* [12]. We and others have previously shown that CDC20 is highly expressed in GSCs compared to non-GSC tumor cells and primary human astrocytes and is required for key GSC phenotypes, including self-renewal and *in vivo* tumorigenicity [12, 17]. However, it is not known if CDC20 also plays a role in GSC treatment resistance [12, 17].

**Fig 1:**
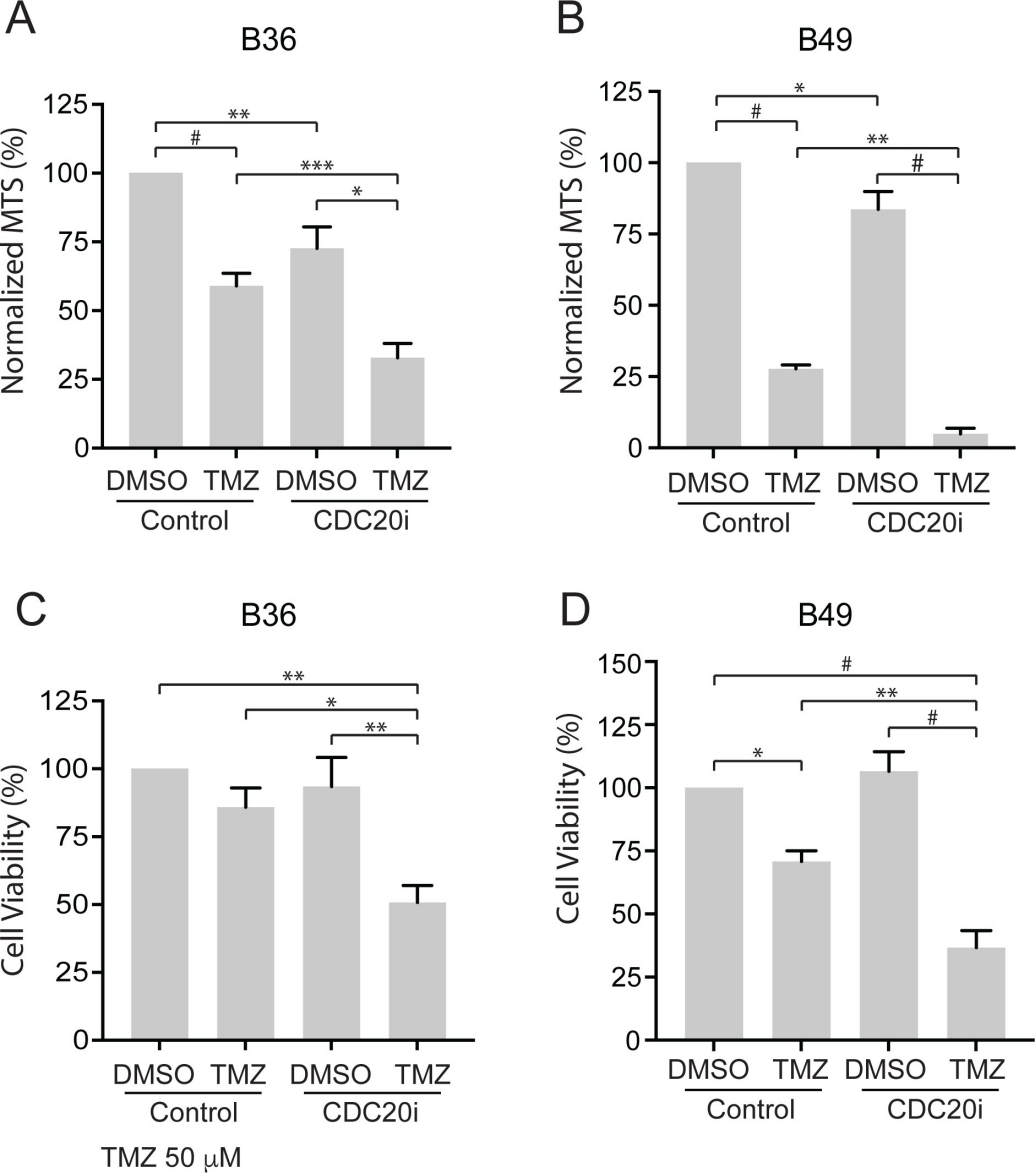
CDC20 inhibition increases sensitivity to TMZ in adherent and neurospheres GSCs. (A) Adherent B36 GSCs were transduced with CDC20 RNAi or control SH002 RNAi and selected with puromycin. Seven days after transduction, GSCs were treated with 50 μM TMZ or vehicle for 10 days. Cell proliferation was then assessed by MTS assay. Data represent mean +/- SEM (n = 7, ANOVA). (B) Adherent B49 GSCs were treated as in (A). Data represent mean +/- SEM (n = 4, ANOVA). (C and D) B36 (C) and B49 (D) GSCs were transduced, passaged to sphere culture format, and treated as in A. 10 days later, cell viability was assessed by luminescent ATP assay. Data represent mean +/- SEM (n = 5, ANOVA). * P < 0.05, ** P < 0.01, *** P < 0.001, # P < 0.0001.

To test whether CDC20 is involved in GSC sensitivity to standard-of-care chemotherapy temozolomide (TMZ), we utilized a lentiviral RNA interference (RNAi) approach to target human *CDC20* using a short hairpin RNA previously shown to efficiently knockdown *CDC20* and, moreover, validated by rescue experiments using CDC20 cDNA resistant to RNAi [12]. We first transduced *CDC20* RNAi (vs. scrambled control RNAi) into human GSCs, treated them with either 50 μM TMZ or vehicle for ten days, and then assessed cell viability by MTS (3-(4,5-dimethylthiazol-2-yl)-5-(3-carboxymethoxyphenyl)-2-(4-sulfophenyl)-2H-tetrazolium) assay for adherent culture or ATP assay for neurosphere culture. TMZ decreased cell viability by 41% in adherent and 14% in neurosphere B36 GSC cultures suggesting 3D sphere cultures are intrinsically more resistant to TMZ than 2D culture (Fig 1A and 1C) [19]. *CDC20* knockdown mildly decreased cell viability in adherent culture and did not significantly decrease cell viability in sphere culture, but increased sensitivity to TMZ, resulting in a 67% and 49% decrease in cell viability in adherent and neurosphere GSC lines, respectively (Fig 1A and 1B). To test the generalizability of CDC20’s role in temozolomide sensitivity, we performed similar experiments in an additional GSC line (B49) and found similar results in both adherent and sphere culture (Fig 1B and 1D). These results suggest that CDC20 promotes temozolomide resistance in GSCs. To demonstrate the specificity of the CDC20 RNAi, we expressed an RNAi-resistant CDC20 cDNA described previously (CDC20-Res) (12) in B36 GSCs in the context of CDC20 RNAi and TMZ treatment (S1A Fig). Expression of CDC20-Res partially but significantly rescued the decreased cell viability triggered by TMZ plus CDC20 RNAi to levels observed with TMZ only.

**Fig 2:**
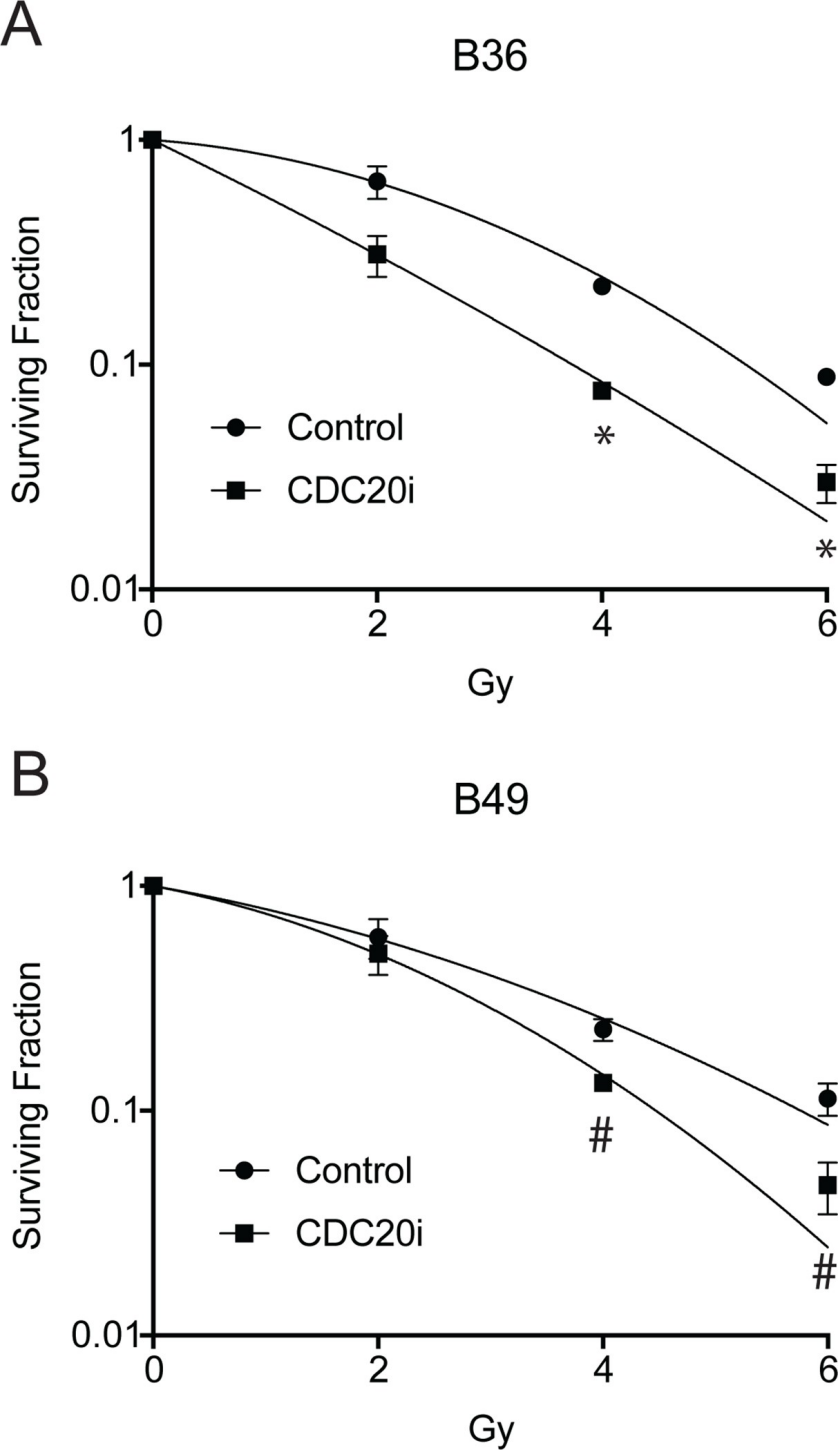
CDC20 inhibition sensitizes GSCs to radiation. (A and B) B36 (A) and B49 (B) GSCs transduced with lentiviruses CDC20 RNAi or control SH002 RNAi and selected with puromycin were plated seven days post-transduction at clonogenic density after puromycin selection. Ionizing radiation (IR) was performed the following day, and clonogenic survival was determined 14 days after IR by staining colonies with 0.5% cresyl violet. The number of colonies was counted to determine the surviving fraction. Data represent mean +/- SEM (unpaired t test). * P < 0.05, ** P < 0.01, *** P < 0.001.

Next, we asked whether CDC20 might also promote GSC radiation resistance. Two GSC lines were exposed to increasing doses of ionizing radiation, and cell survival was measured 10 days later by clonogenic assay. *CDC20* knockdown sensitized cells to ionizing radiation in a dose-dependent manner (Fig 2A and 2B). To show the specificity of the CDC20 RNAi in this context, we expressed CDC20-Res in the setting of CDC20 RNAi in B36 GSCs and subjected them to ionizing radiation (S1B Fig). Transduction of CDC20-Res rescued the decrease in colony counts induced by both CDC20 RNAi and ionizing radiation to levels seen with ionizing radiation alone. Together, these results indicate that CDC20 promotes resistance to both TMZ and ionizing radiation and that CDC20 inhibition can sensitize GSCs to standard of care therapies.

**Fig 3:**
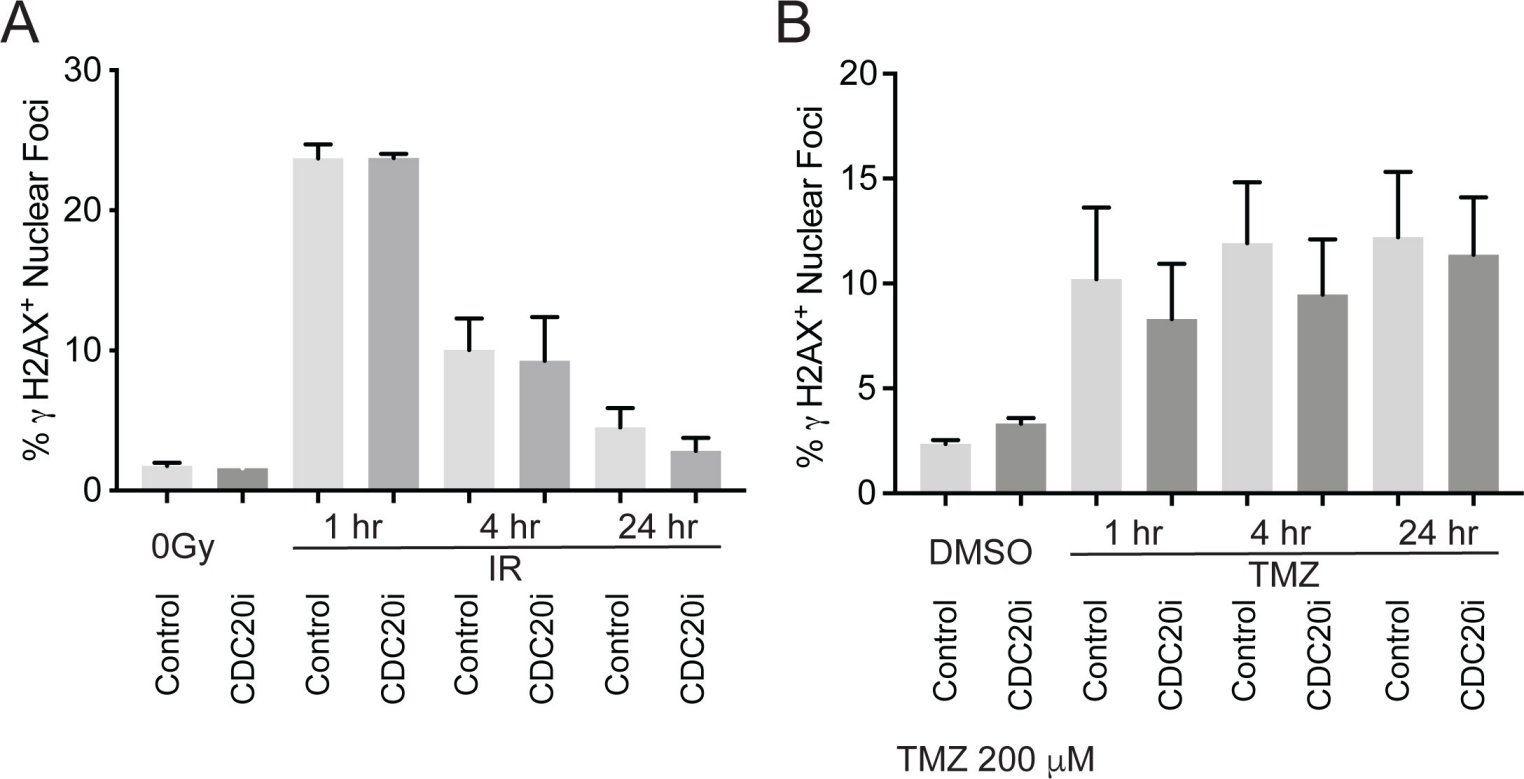
CDC20 knockdown does not affect DNA damage following TMZ or radiation treatment. (A) B36 GSCs transduced with lentiviruses CDC20 RNAi or control SH002 RNAi were selected with puromycin and plated at the indicated density 7 days post-transduction, and then treated with 2 Gy IR the following day. Cells were then processed for γ-H2AX immunofluorescence at the indicated time points. Data represent mean +/- SEM (n = 3). (B) B36 GSCs transduced and plated as in (A) were treated with 200 μM TMZ the following day. Cells were then processed for γ-H2AX immunofluorescence at the indicated time points. Data represent mean +/- SEM (n = 3).

To investigate the cellular mechanism of how CDC20 affects sensitivity to temozolomide and radiation, we first considered the possibility that CDC20 inhibition may sensitize cells to DNA damage or interfere with DNA damage repair. We transduced GSCs with *CDC20* RNAi versus scrambled control RNAi and then treated them with ionizing radiation or TMZ. DNA damage was assessed 1, 4, and 24 hours after treatment by counting γ-H2AX^+^ nuclei foci [20]. GSCs have been shown to have intrinsic radioresistance through activation of the DNA damage checkpoint [8]. In line with prior reports, we found that the number of γ-H2AX^+^ foci per cell peaked 1 hour after ionizing radiation and then progressively returned to baseline levels by 24 hours [8, 9]. γ-H2AX^+^ foci after TMZ increased at 1 hour and remained unchanged at 4 and 24 hours. *CDC20* knockdown did not significantly alter radiation- or TMZ- induced DNA damage at any of the timepoints (RT: *P* > 0.999, TMZ *P* > 0.999 for comparisons at each timepoint (ANOVA, Bonferroni), Fig 3A and 3B). These results suggest that CDC20 inhibition does not obviously sensitize cells to DNA damage or interfere with DNA damage repair.

**Fig 4:**
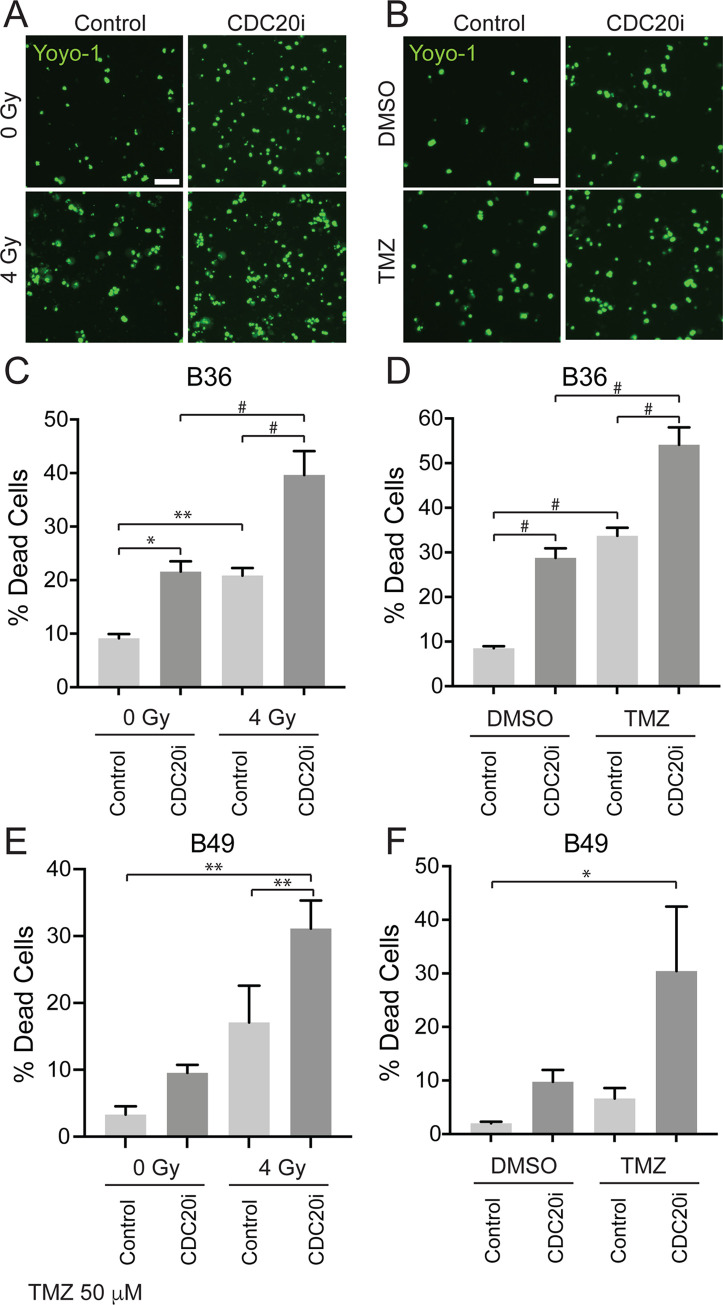
CDC20 inhibition increased cell death following treatment with TMZ or radiation. (A) B36 GSCs transduced with indicated lentivirus were selected with puromycin and plated at the indicated density 7 days post-transduction, and then treated with 4 Gy IR the following day. 24 hours after IR, Cells were then labeled with YOYO-1 fluorescent dye for 6 days. (B) GSCs transduced and plated as in (A), were treated with 200 μM TMZ the following day for 24 hours. Cells were then labeled with YOYO-1 as in (A). (C) Quantification of YOYO-1 positive cells from (A). Data represent mean +/- SEM (n = 3, ANOVA). (D) Quantification of YOYO-1 positive cells from (B). Data represent mean +/- SEM (n = 3, ANOVA). (E) B49 GSCs were treated as in (A) and dead cells quantified as in (C). Data represent mean +/- SEM (n = 4, ANOVA). (F) B49 GSCs were treated as in (B) and dead cells quantified as in (C). Data represent mean +/- SEM (n = 4, ANOVA).* P < 0.05, ** P < 0.01, # P < 0.0001.

**Fig 5:**
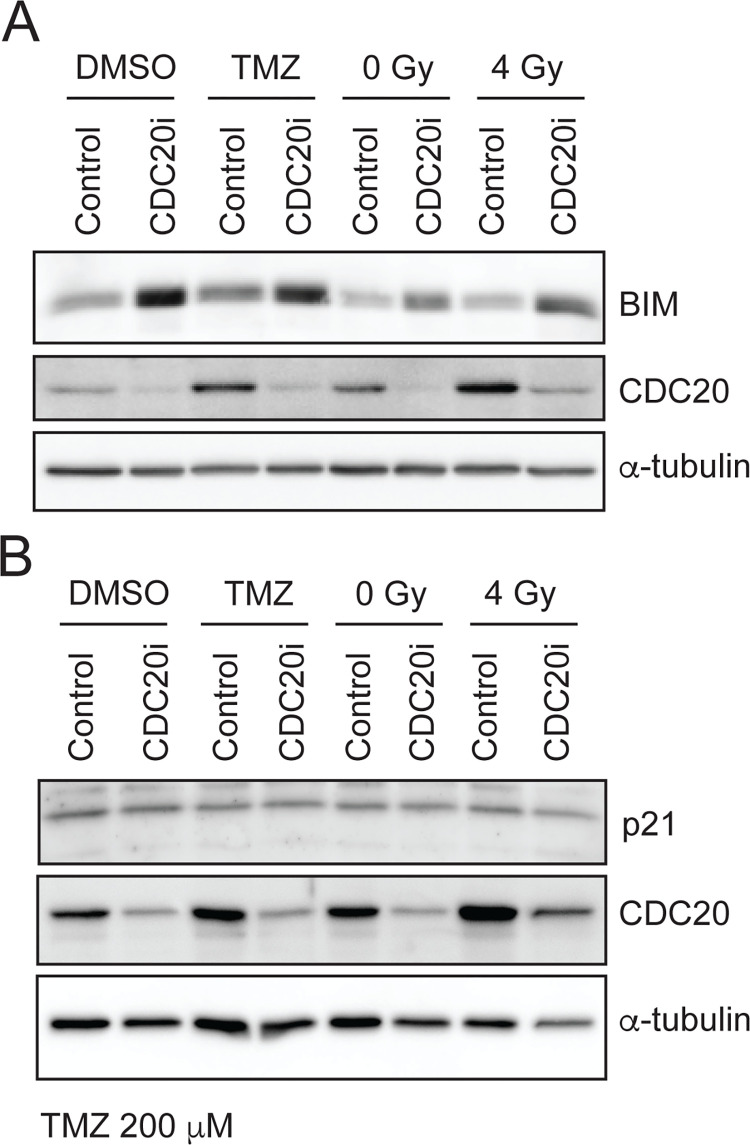
CDC20 inhibition is associated with increased Bim protein in the setting of TMZ and ionizing radiation. (A) B36 GSCs transduced with CDC20 RNAi or a non-targeting control and selected with puromycin were re-plated and then treated the following day with 4 Gy IR or 200 μM TMZ for 24 hours. Protein lysates were then harvested and analyzed by immunoblotting with the indicated antibodies. (B) B36 GSCs were treated and analyzed as in (A).

Unrepaired DNA damage caused by radiation or TMZ typically induces either senescence or cell death [21, 22]. To test if CDC20 inhibition increases radiation- or TMZ-induced cell death, GSCs were transduced with *CDC20* RNAi versus scrambled control RNAi and treated with ionizing radiation or TMZ. Cells were then exposed to YOYO-1, a fluorescent dye that labels DNA. Living cells with intact plasma membranes are impermeable to YOYO-1, whereas dead cells with disrupted plasma membranes will exhibit nuclear staining. As expected, radiation and TMZ increased cell death to 21% and 34%, respectively (Fig 4A–4D). Although *CDC20* knockdown alone mildly increased cell death, *CDC20* knockdown markedly enhanced cell death when combined with radiation treatment or TMZ, resulting in 40% and 54% cell death, respectively (Fig 4A–4D). Similar results were observed in a second GSC line (Fig 4E and 4F). Although smaller differences were observed among some conditions in B49 cells, the combination of CDC20 RNAi plus either ionizing radiation or TMZ robustly increased cell death compared to control-treated cells. In other cell contexts, CDC20-APC is known to govern cellular apoptosis by ubiquitinating and thus destabilizing pro-apoptotic proteins, such as Bim (BCL2L11) [23]. We thus hypothesized that CDC20 RNAi might be increasing GSC sensitivity to therapy through this mechanism. In line with this, immunoblotting of protein lysates harvested from control GSCs showed low baseline levels of Bim, which increased following *CDC20* knockdown, regardless of treatment (Fig 5A). Another CDC20 substrate, p21, which has a significant role in cell cycle control [24], was also examined in the context of CDC20 RNAi and both TMZ and ionizing radiation, but we found that p21 levels did not significantly change with CDC20 RNAi in the context of either TMZ or ionizing radiation (Fig 5B). This suggests that *CDC20* knockdown leads to Bim accumulation, shifting the cell into a pro-apoptotic state and promoting cell death in response to radiation and TMZ-induced DNA damage.

## Discussion

**Fig 6:**
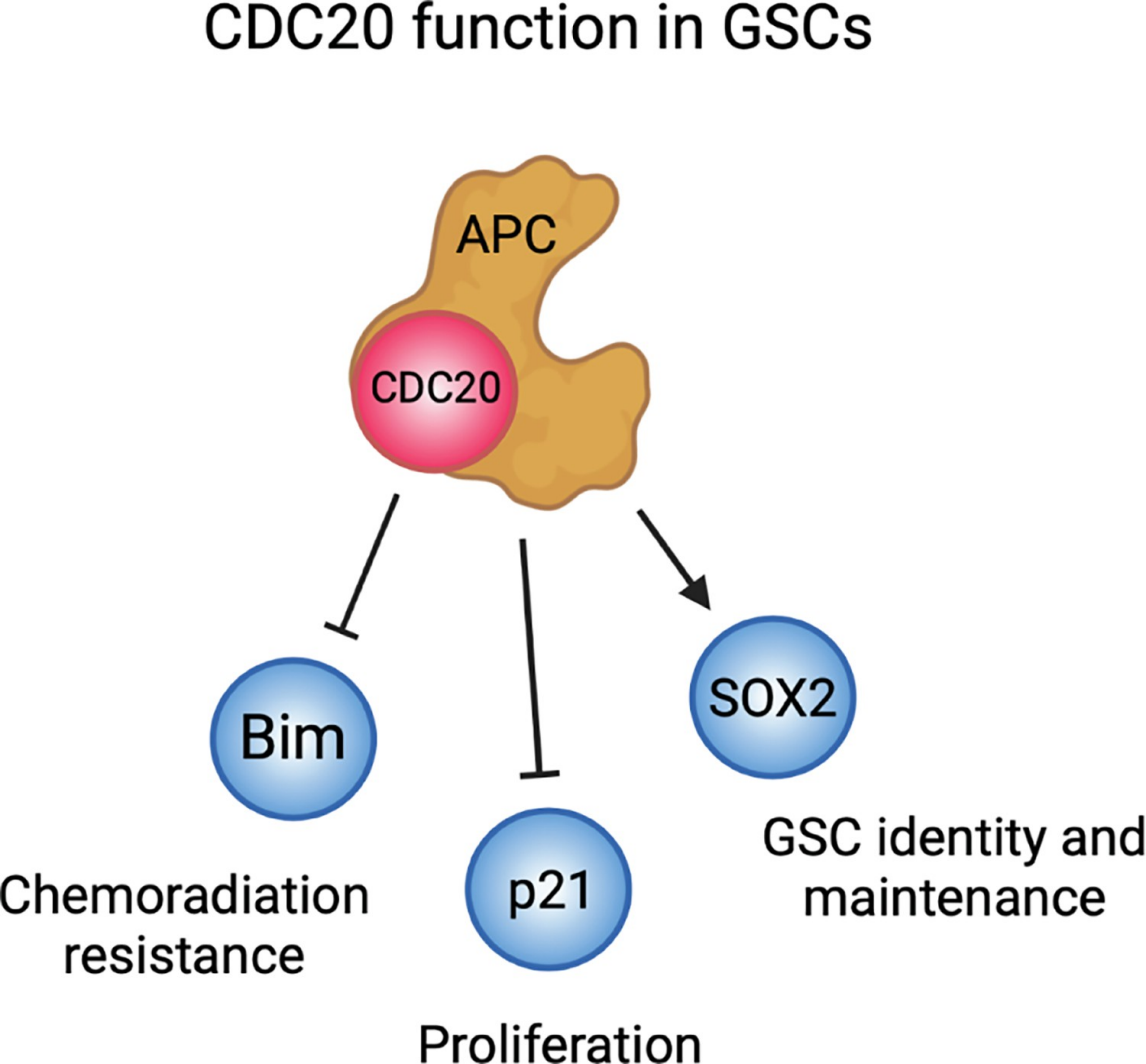
Mechanisms of CDC20-APC in GSCs. The current study identifies a role for CDC20-APC substrate Bim in GSC chemoradiation resistance. Other relevant substrates of CDC20-APC in GSCs include p21 (17) and SOX2 (12). Schematic created using Biorender.com.

In this study, we have shown, through a loss-of-function approach, that CDC20 promotes resistance to TMZ and radiation in GSCs. CDC20 knockdown does not appear to increase TMZ- or RT-mediated DNA damage per se but instead promotes cell death through accumulation of the pro-apoptotic protein, Bim. In the context of other roles for CDC20 in GSCS, these findings suggest a specific pathway for CDC20 in therapeutic resistance (Fig 6).

Resistance to standard of care TMZ and radiation is a key feature of glioblastoma, and is in large part responsible for inevitable recurrence. GSCs represent a distinct cell population within GBM tumors, which may drive tumor recurrence and treatment failure [8, 10]. Chen et al. identified a population of tumor initiating cells capable of generating GBM tumors *in vivo* following treatment with TMZ [10]. Bao et al. showed that upregulation of the DNA damage response in GSCs, as defined by CD133 expression, results in increased resistance to ionizing radiation compared to non-GSC cells [8]. We have previously demonstrated that CDC20 is a critical determinant of GSC phenotypes, specifically invasiveness, self-renewal, and tumor growth [12, 17]. Other pathways implicated in maintaining GSC stemness have been manipulated to increase radio- and chemo-sensitivity [25–27]. While a possible role for CDC20 in treatment resistance has previously been explored in head and neck cancers [23], our results are the first to suggest a direct and causal link between CDC20 and radio- and chemoresistance in GSCs.

To further elucidate how CDC20 affects sensitivity to TMZ and radiation, we performed a temporal quantification of γ-H2AX foci in GSCs after treatment with TMZ or radiation. γ-H2AX is a sensitive marker of double stranded DNA breaks (DSB) and is observed following treatment with radiation or TMZ [28, 29]. γ-H2AX is also important in DNA damage checkpoint control, triggering DSB repair [20]. Preferential activation of the DNA damage response has been identified as an important driver of radio- and chemoresistance in GSCs, and selective inhibition of different DNA damage response pathways increases sensitivity to these treatments [8, 9, 25, 26, 30]. Our results show that DNA damage repair is not overtly affected by CDC20 knockdown. This is not unexpected, given that the CDC20-APC complex functions primarily in mitosis, whereas the G1/S-associated CDH1-APC complex has been implicated in DNA damage repair [31].

In addition to its role in cell cycle regulation [13], CDC20 is known to regulate apoptosis through the ubiquitination and destabilization of pro-apoptotic proteins, such as Bim [32]. Wan et al. previously demonstrated that Bim is a direct substrate of CDC20-APC [23]. Knockdown of CDC20 in different cancer cell lines with high baseline CDC20 expression resulted in upregulation of Bim and increased sensitivity to radiation and a number of chemotherapeutics [23]. Our study is the first to demonstrate this relationship between CDC20 and Bim in primary human cancer cells. CDC20 knockdown and subsequent accumulation of Bim may shift GSCs into a pro-apoptotic state, promoting cell death in response to TMZ- and radiation-induced DNA damage before DNA damage repair mechanisms can be adequately activated. However, we cannot fully exclude the possibility that cell cycle perturbations or other reported CDC20-APC mechanisms, such as protection of SOX2 protein (12), may be also be underlying some of the effects of combined CDC20 inhibition plus chemo- or radiation therapy, a direction that should be pursued in future studies.

CDC20 is viewed as a promising therapeutic target in multiple cancers [32]. CDC20 expression is elevated in glioblastoma compared to low-grade gliomas, and patients with high CDC20-expressing GBMs in the Proneural subtype have significantly shorter overall survival [12]. This may in part be due to CDC20-mediated increased invasive potential and self-renewal [12]. Our current study suggests that CDC20 may also affect survival by increasing resistance to standard of care TMZ and radiation therapy. Therapeutic strategies to improve the efficacy of existing treatment for GBM are desperately needed. Perturbation of the DNA damage response has received significant attention as both a cancer driver and therapeutic target to increase radio- and chemosensitivity in GSCs [8, 9, 25, 26, 30, 33, 34]. Here we show that CDC20-dependent regulation of apoptosis represents another promising therapeutic target. Future studies are necessary to elucidate the precise mechanisms of CDC20-mediated radio- and chemoresistance, including *in vivo* validation using small molecule CDC20 inhibitors.

## Supporting information

S1 Fig CDC20 RNAi phenotypes can be rescued by co-expression of RNAi-refractory CDC20. (A) B36 GSCs transduced with CDC20 RNAi viruses as in Fig 1A were transduced two days later with RNAi-resistant CDC20-expressing lentiviruses, selected with puromycin for 5 days, and treated with TMZ as in Fig 1A. Control = SH002 + N103 vectors. Vec = N103. Ten days later, cell viability was assessed by luminescent ATP assay. Data represent mean +/- SEM (n = 3, ANOVA). * P < 0.005, ** P < 0.001. (B) B36 GSCs transduced with CDC20 RNAi viruses as in Fig 2A were transduced two days later with RNAi-resistant CDC20-expressing lentiviruses, selected with puromycin for 5 days, and then treated as in Fig 2A Control = SH002 + N103 vectors. Vec = N103. 4 Gy of IR was used. 10 days later, colony counts were performed and normalized to Control. Data represent mean +/- SEM (n = 5, ANOVA). * P < 0.02.
(PDF)

S1 Raw images (PDF)
