# Supplementary figures and images for: CDC20 regulates sensitivity to chemotherapy and radiation in glioblastoma stem cells
Source: PLoS One. 2022 Jun 23;17(6):e0270251. doi: 10.1371/journal.pone.0270251 (PMC9223386; doi:10.1371/journal.pone.0270251)

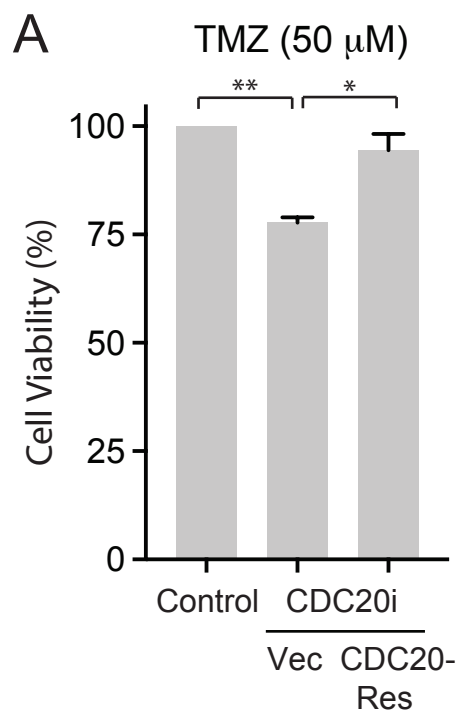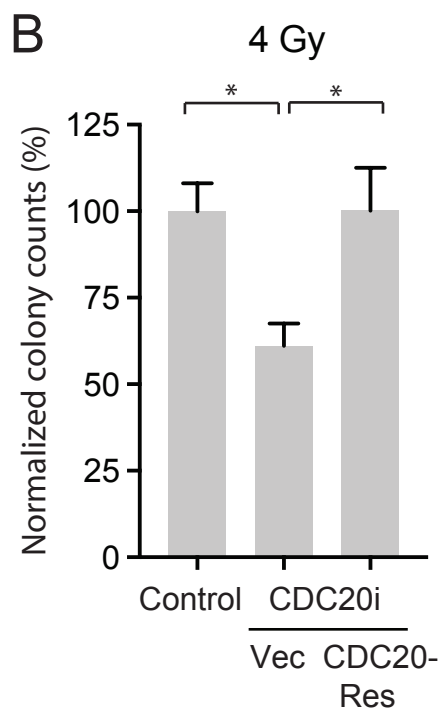

Supplement: S1 Fig — (A) B36 GSCs transduced with CDC20 RNAi viruses as in Fig 1A were transduced two days later with RNAi-resistant CDC20-expressing lentiviruses, selected with puromycin for 5 days, and treated with TMZ as in Fig 1A. Control = SH002 + N103 vectors. Vec = N103. Ten days later, cell viability was assessed by luminescent ATP assay. Data represent mean +/- SEM (n = 3, ANOVA). * P < 0.005, ** P < 0.001. (B) B36 GSCs transduced with CDC20 RNAi viruses as in Fig 2A were transduced two days later with RNAi-resistant CDC20-expressing lentiviruses, selected with puromycin for 5 days, and then treated as in Fig 2A Control = SH002 + N103 vectors. Vec = N103. 4 Gy of IR was used. 10 days later, colony counts were performed and normalized to Control. Data represent mean +/- SEM (n = 5, ANOVA). * P < 0.02. (PDF) [file pone.0270251.s001.pdf]
