# Supplementary material for: CDC20 regulates sensitivity to chemotherapy and radiation in glioblastoma stem cells
Source: PLoS One. 2022 Jun 23;17(6):e0270251. doi: 10.1371/journal.pone.0270251 (PMC9223386; doi:10.1371/journal.pone.0270251)

Fig 5A
anti-BIM immunoblot

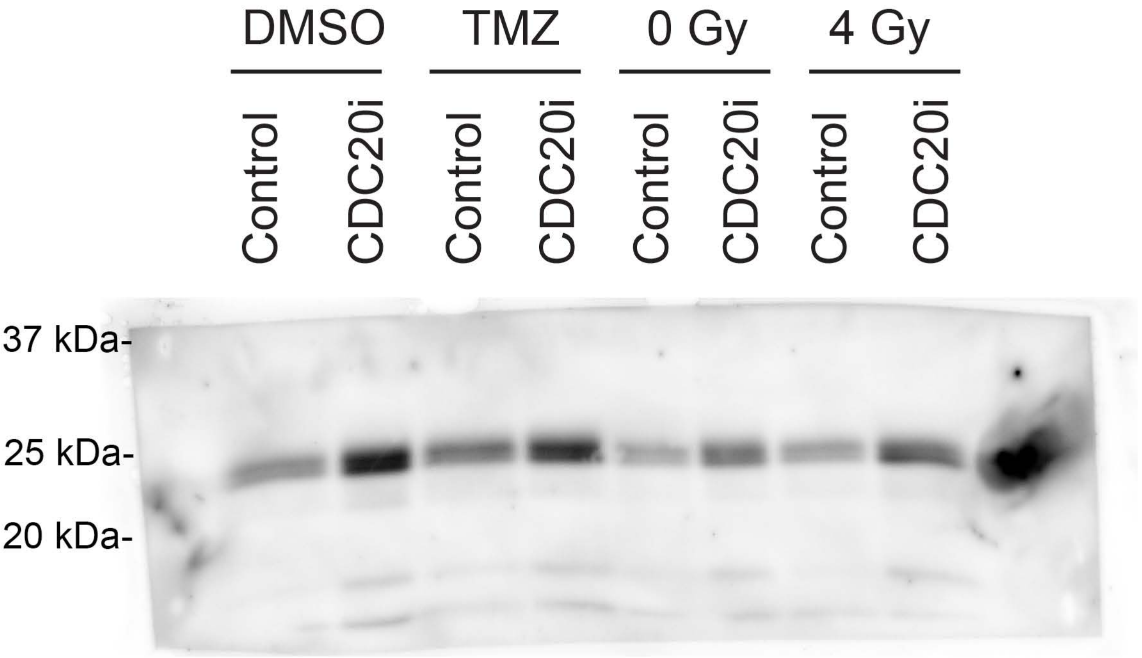

# Fig 5A
anti-CDC20 immunoblot

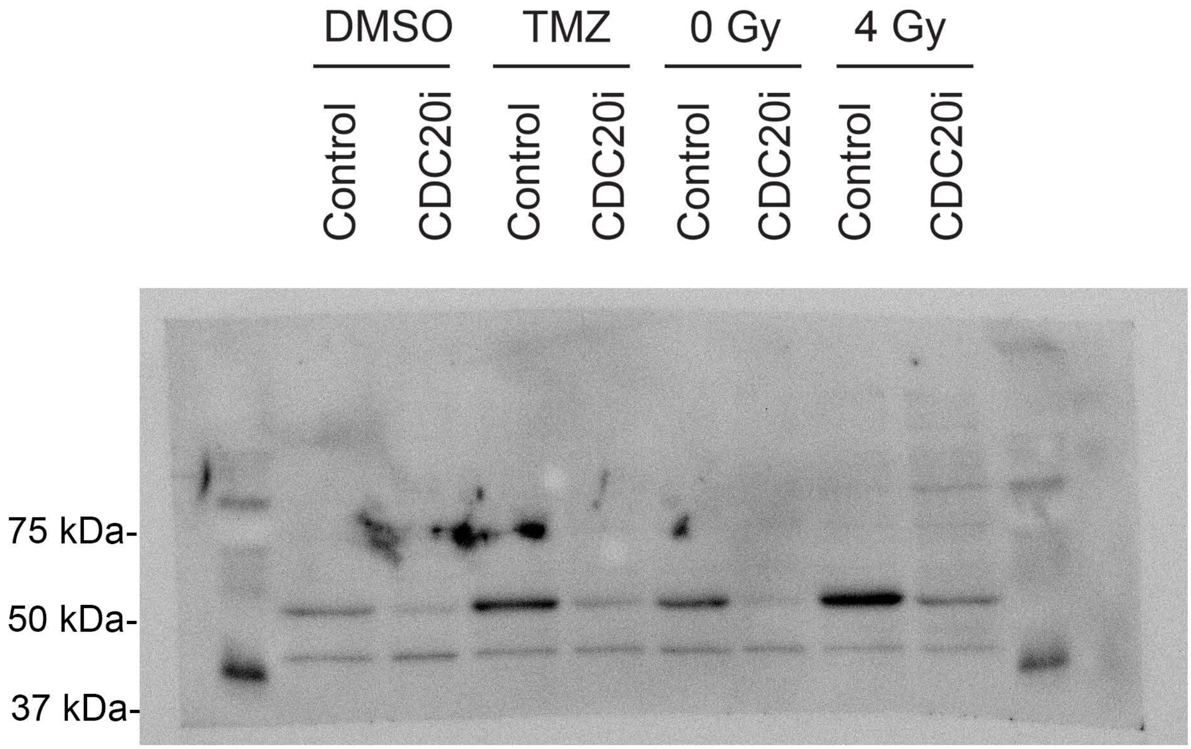

Fig 5A
anti-alpha-tubulin immunoblot

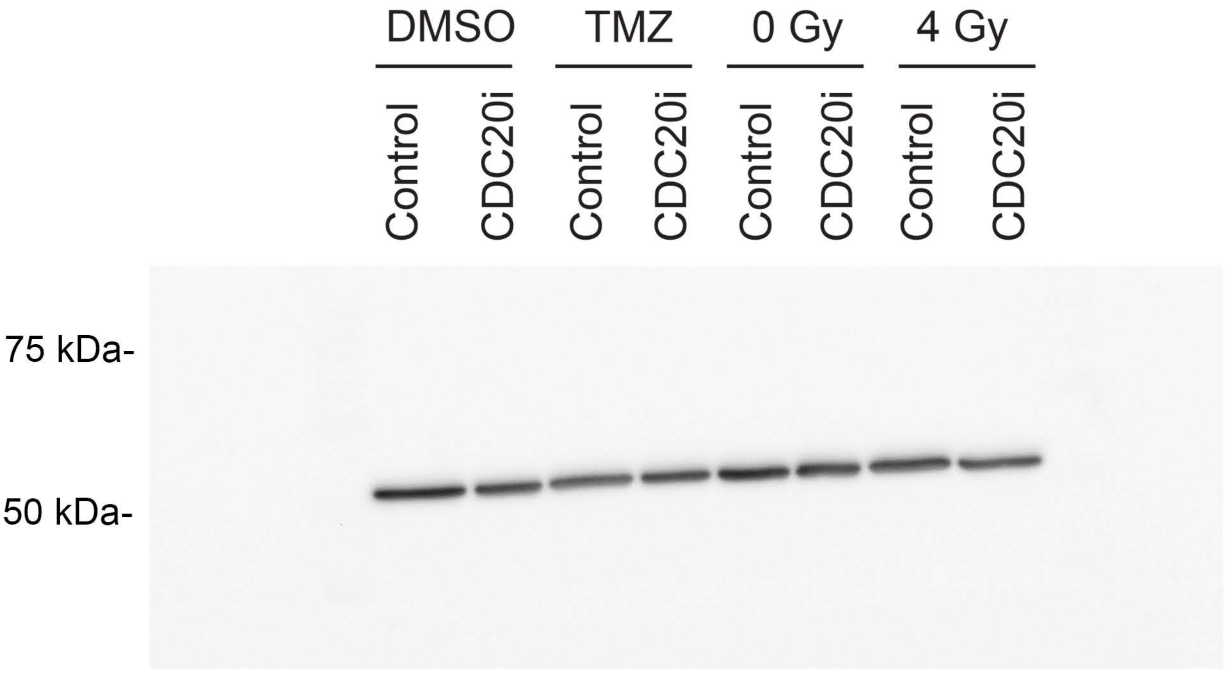

# Fig 5B
anti-p21 immunoblot

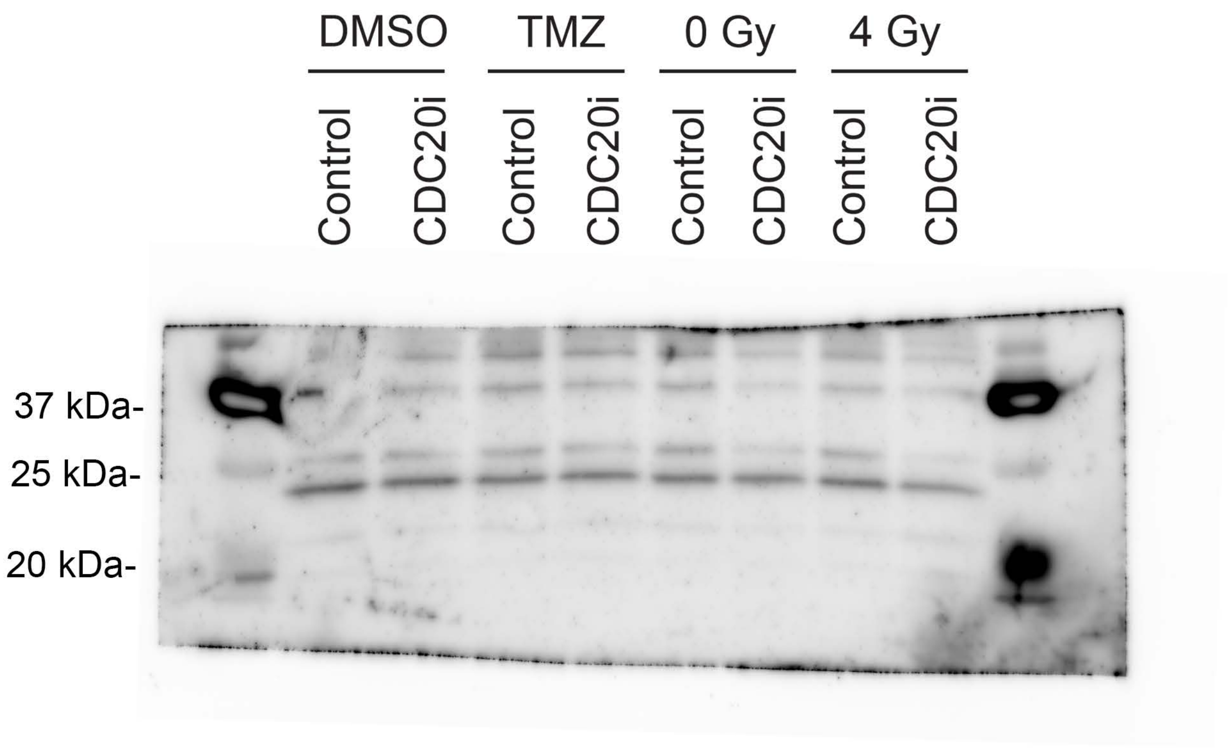

# Fig 5B
anti-CDC20 immunoblot

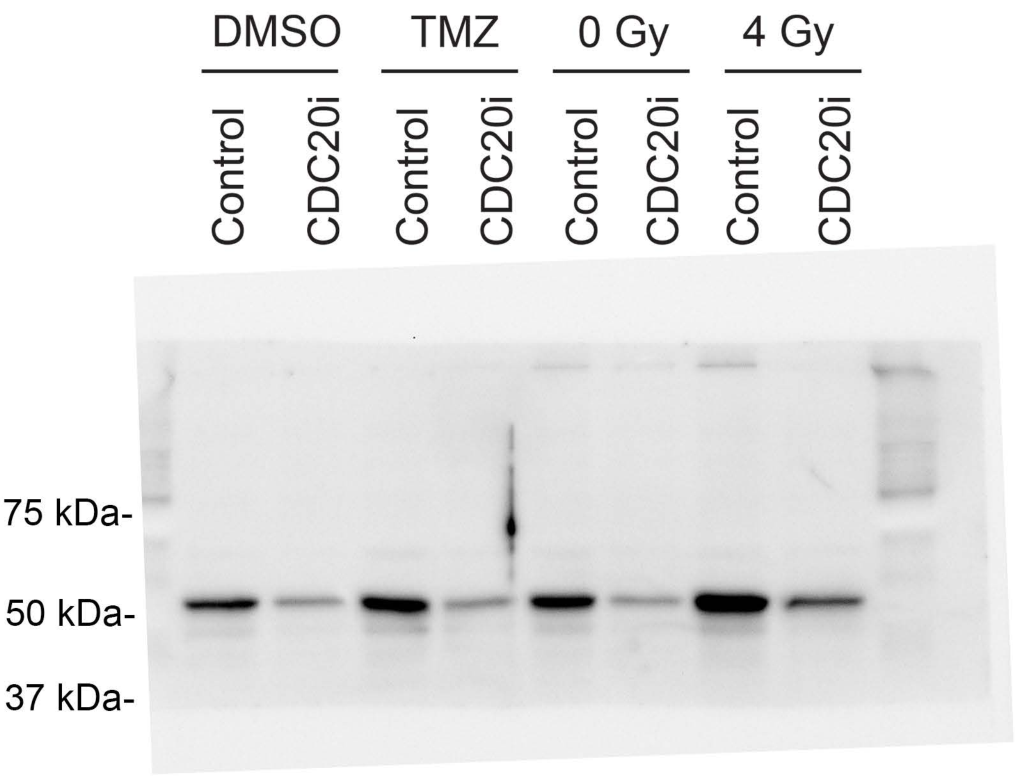

Fig 5B
anti-alpha-tubulin immunoblot

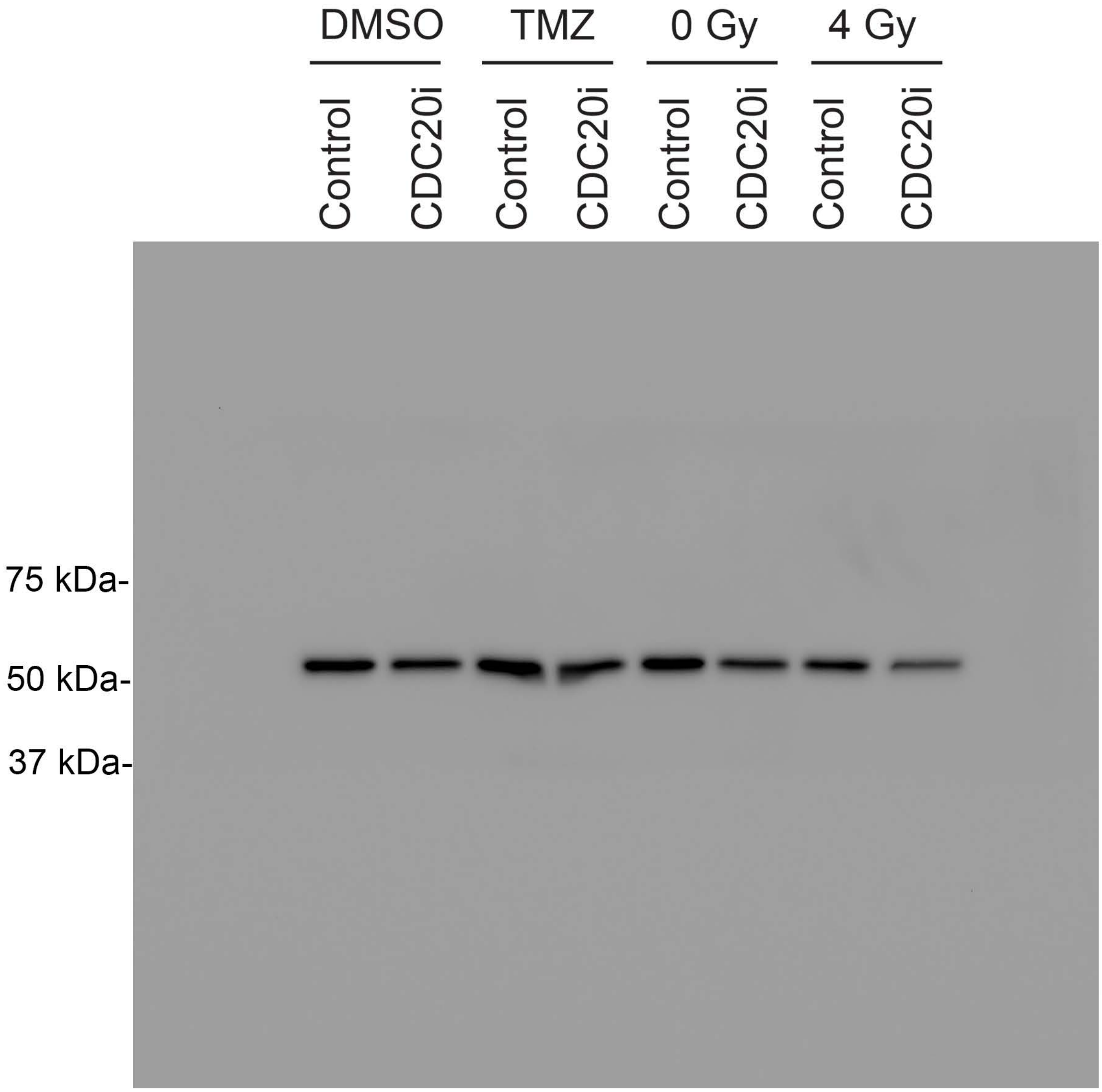

Supplement: S1 Raw images — (PDF) [file pone.0270251.s002.pdf]
